# Hyperthermophilic endospores germinate and metabolize organic carbon in sediments heated to 80°C

**DOI:** 10.1111/1462-2920.16167

**Published:** 2022-09-13

**Authors:** Emma Bell, Jayne E. Rattray, Kathryn Sloan, Angela Sherry, Giovanni Pilloni, Casey R. J. Hubert

**Affiliations:** ^1^ Geomicrobiology Group, Department of Biological Sciences University of Calgary Calgary Alberta Canada; ^2^ School of Natural and Environmental Sciences Newcastle University Newcastle upon Tyne UK; ^3^ Hub for Biotechnology in the Built Environment, Department of Applied Sciences Northumbria University Newcastle upon Tyne UK; ^4^ ExxonMobil Technology and Engineering Company Annandale New Jersey USA

## Abstract

Cold surface sediments host a seedbank of functionally diverse thermophilic bacteria. These thermophiles are present as endospores, which are widely dispersed in aquatic environments. Here, we investigated the functional potential of endospore populations in cold surface sediments heated to 80°C. Microbial production of acetate was observed at 80°C and could be enhanced by supplying additional organic carbon substrates. Comparison of 16S rRNA gene amplicon libraries from 80°C enrichments to sediments heated to lower temperatures (50–70°C) showed that temperature selects for distinct populations of endospore‐forming bacteria. Whereas sulfate‐reducing thermophiles were enriched in 50–70°C incubations, 80°C exceeds their thermal tolerance and selects for hyperthermophilic organotrophic bacteria that are similarly detected in amplicon libraries from sediments heated to 90°C. Genome‐resolved metagenomics revealed novel carbon cycling members of *Symbiobacteriales*, *Thermosediminibacteraceae*, *Thermanaeromonas* and *Calditerricola* with the genomic potential for the degradation of carbohydrates, sugars, amino acids and nucleotides. Endospores of thermophilic bacteria are deposited on seabed sediments worldwide where they remain dormant as they are buried in the accumulating sediments. Our results suggest that endospore populations could be activated by temperature increases encountered during burial and show the potential for organotrophic metabolic activity contributing to acetate generation in deep hot sediments.

## INTRODUCTION

Over one‐third of marine sediments globally are heated above 60°C, and one‐quarter are above 80°C (LaRowe et al., 2017); yet, little is known about microbial populations that reside in deep, hot sediments. As sediment temperature increases with depth, microbial cell numbers are in decline, consistent with biomass generally diminishing with depth owing to decreasing energy availability over time (Kallmeyer et al., 2012). Low biomass in deep, hot sediments makes direct sequencing of microbial DNA challenging (Dombrowski et al., 2018; Heuer et al., 2020), thereby limiting understanding of extant deep subsurface populations.

Experiments that employ heating of surface sediments provide an opportunity to explore temperature‐dependent effects on sediment chemistry associated with resident microbial populations. In experiments designed to mimic heating‐during‐burial using both surface and subseafloor sediments, acetate production is observed (Parkes et al., 2007; Roussel et al., 2015; Wellsbury et al., 1997, 2002). This mirrors observations of deep, hot sediments in situ where acetate accumulation has also been reported (Egeberg & Barth, 1998; Heuer et al., 2020; Wellsbury et al., 1997) that suggest acetate is an important energy source in the deep hot biosphere.

Sediment heating studies have also shown that endospores of thermophilic bacteria are widespread in cold surface environments where they cannot grow (Hubert et al., 2009; Müller et al., 2014). These dormant endospore populations are deposited on the seafloor from the water column, following a period of passive dispersal in ocean currents (de Rezende et al., 2013; Nielsen et al., 2017; Volpi et al., 2017). Once deposited, thermophilic endospores remain dormant as they are buried in the accumulating sediments. Dormancy offers a survival strategy for populations adapted to warmer temperatures to persist during burial through cooler, shallower intervals. As such, thermophilic endospores have been recovered from subseafloor sediments ranging from 4500 years old (de Rezende et al., 2013) to 20‐million years old (Fang et al., 2017), showing that endospores in deeply buried sediments remain viable.

Increasing temperature during burial could provide an opportunity for dormant thermophilic endospores to germinate. In agreement with this, previously reported sediment heating experiments have shown that different temperatures apply selective pressure that results in germination and growth of different dormant endospore populations (Bell et al., 2020; de Rezende et al., 2013). In those studies, incubating sediment at temperatures between 45 and 70°C resulted in the enrichment of distinct populations of sulfate‐reducing bacteria, whereas sulfate reduction was not observed at ≥80°C. This apparent cut‐off has also been postulated for microorganisms in sedimentary environments, inferred from observations that anaerobic hydrocarbon biodegradation has not occurred in oil reservoir formations buried to depths hotter than 80°C (Head et al., 2003; Wilhelms et al., 2001). On the other hand, the upper temperature limit for life in deeply buried marine sediments has recently been shown to extend up to at least 120°C, with isotope data (Heuer et al., 2020) and radiotracer experiments (Beulig et al., 2022) suggesting deeply buried hot sediments host acetate‐utilizing hyperthermophiles. This means that populations in the hyperthermophilic temperature range are not necessarily restricted to hydrothermal systems at mid‐oceanic ridges where reduced inorganic compounds support chemoautotrophs with the highest known growth temperatures (Kashefi & Lovley, 2003; Takai et al., 2008).

During experiments testing the thermal tolerance of endospore‐forming sulfate‐reducing bacteria, we observed acetate production in sediments heated to 80°C. It was expected that sulfate‐reducing bacteria would produce acetate from the incomplete oxidation of organic electron donors in cooler incubations, but 80°C exceeded the thermal tolerance of sulfate‐reducing bacteria (Bell et al., 2020). This study therefore tests the hypothesis that sediments host populations of hyperthermophilic endospores that can be stimulated to catalyse organic matter degradation upon heating to 80°C.

## EXPERIMENTAL PROCEDURES

### Preparation of heated sediment slurries

Sediment from the Tyne estuary, United Kingdom (54°57′51″ N, 1°40′60″ W) was used as inoculum of endospores in all sediment heating experiments. Sediment was collected at low tide with a trowel to ~20 cm depth and stored in sealed plastic containers at 4°C. Sediment collected in 2013 and 2017 was used for experiments performed 2014 and 2022. Anoxic sediment slurries were prepared by mixing sediment and anoxic seawater medium at a fixed ratio (1 g sediment to 2 ml medium) under a constant flow of N_2_ (Isaksen et al., 1994; Widdel & Bak, 1992). For experiments supplemented with organic carbon, tryptic soy broth (3 g/L), glucose (3 mM) and the carboxylic acids acetate, propionate, butyrate and lactate (3 mM each) were added from sterile stock solutions. Glass serum bottles containing sediment slurries (50 ml) were sealed with a butyl rubber stopper and incubated at 50, 60, 70, 80 and 90°C. Sterilized controls were prepared by the addition of zinc chloride into the slurries to a final concentration of 10% prior to incubation at 80°C.

During the incubation period, heated anoxic sediment slurries were subsampled (1.5 ml) with an N_2_ flushed syringe. Subsamples were centrifuged (13,000 g, 5 min) with the resulting supernatant used to measure organic acids and the sediment pellet used for DNA extraction.

### Organic acid measurements

Sediment pore water organic acids were measured using two methods: ion (exclusion) chromatography (IC) or high‐performance liquid chromatography (HPLC). Samples measured by IC were filtered through 0.45 μm Teflon filters and acidified with 0.1 M octanesulfonic acid (1:1). Acidified samples were sonicated for 30 minutes to remove bicarbonate as CO_2_. Acetate, propionate and butyrate were measured in the acidified samples by ion (exclusion) chromatography (IC) using a Dionex ICS‐1000 with an AS40 auto‐sampler equipped with an IonPac ICE‐AS1, 4 × 250 mm analytical column. The IC flow rate was 0.16 ml/min, the eluent was 1.0 mM heptafluorobutyric acid and the cation regenerant solution used for the AMMS‐ICE II supressor was 5 mM tetrabutylammonium hydroxide. Samples measured by HPLC were filtered through 0.20 μm polytetrafluoroethylene (PTFE) filters. Formate, acetate, propionate, lactate, butyrate and succinate were measured using UV (210 nm) on an HPLC RSLC Ultimate 3000 following the method previously described by Volpi et al. (2017) for heated sediment incubations. Briefly, organic acids were separated using an Aminex HPX‐87H, 7.8 × 300 mm analytical column using 5 mM H_2_SO_4_ as the isocratic eluent, a flow rate of 0.6 ml/min, and the column oven was heated to 60°C.

### DNA extraction and 16S rRNA gene amplicon sequencing

DNA was extracted from sediment pellets using the PowerSoil DNA isolation Kit (MoBio Laboratories) following the manufacturer's protocol, except for the elution step, which was modified by adding 50 μl elution buffer and allowing 30 min before eluting by centrifugation. Extracted DNA was used as a template for PCR amplification using Golay barcoded fusion primers targeting the V4–V5 region of the 16S rRNA gene (Caporaso et al., 2012). The PCR protocol included denaturation at 95°C for 4 min followed by 25 cycles consisting of denaturation (1 min, 95°C), annealing (45 s, 55°C) and extension (1 min, 72°C) and a final extension for 10 min at 72°C. PCR products derived from a common sub‐sampling time from triplicate sediment slurries were in most instances pooled prior to clean‐up using Agencourt Ampure XP paramagnetic beads resulting in a single pooled amplicon library for a given experimental time point. 16S rRNA gene amplicons were sequenced on an Ion Torrent Personal Genome Machine (School of Natural and Environmental Sciences, Newcastle University, UK) in accordance with the manufacturer's instructions (Life Technologies). Sequencing data were processed by the Torrent Suite Software V4.0. Raw sequence reads were demultiplexed and quality filtered in QIIME version 1.9.1 (Caporaso et al., 2010). All subsequent sequence analysis was performed with USEARCH v11 (Edgar, 2013). Sequences were truncated to 350 bp (*fastx_truncate*) and clustered into operational taxonomic units (OTUs) sharing 97% sequence identity with UPARSE (*cluster_otus*). Taxonomy was predicted with SINTAX (Edgar, 2016) with a USEARCH compatible (Lee, 2020) Silva 138 database (Quast et al., 2013). Amplicon data were visualized with the R package Ampvis2 (Andersen et al., 2018). Normalized OTU counts were used to calculate the correlations between each OTU and the concentration of organic acids at the corresponding sampling time points using the Python library Pandas (The Pandas Development Team, 2021).

### Metagenomic sequencing, assembly, binning and analyses

Metagenomic sequencing was performed on an Illumina NovaSeq 6000 with a S4 300 cycle flow cell. Libraries were prepared by shearing to an insert size of ~200 bp using a Covaris instrument, followed by library construction with the NEB Ultra II DNA library prep kit. Reads were pre‐processed with BBDuk (Bushnell et al., 2017) and assembled separately with two assemblers (1) metaSPAdes (Nurk et al., 2017) and (2) MEGAHIT (Li et al., 2015) using the *meta‐sensitive* option. Raw reads were mapped to each of the assemblies with BBMap (Bushnell et al., 2017). Each of the assemblies was binned with both MetaBAT2 (Kang et al., 2019) and CONCOCT (Alneberg et al., 2014). Bins from the same assembler were refined using DAS Tool (Sieber et al., 2018). The best bins from each of the approaches were selected with dRep (Olm et al., 2017) using the parameters; completeness 75%, contamination 5%, primary cluster average nucleotide identity (ANI) 90%, secondary cluster ANI 99%. This resulted in a total of 13 representative metagenome assembled genomes (MAGs).

Protein‐coding genes were predicted with prodigal (Hyatt et al., 2010). Amino acid sequences used to predict the optimum growth temperature of microorganisms using Tome (Li et al., 2019). MAGs from microorganisms predicted to be thermophiles were annotated with METABOLIC v4.0 (Zhou et al., 2022), which integrates annotation of protein‐coding genes with KOfam (Aramaki et al., 2020), TIGRfam (Selengut et al., 2007), Pfam (Finn et al., 2014), dbCAN2 (Zhang et al., 2018) and MEROPS (Rawlings et al., 2016). 16S rRNA gene sequences in the metagenomic dataset were identified with METAXA2 (Bengtsson‐Palme et al., 2015) and phyloFlash (Gruber‐Vodicka et al., 2020). 16S rRNA sequences from the metagenomic dataset were aligned to 16S rRNA gene amplicon sequences with BLAST (Altschul et al., 1990).

MAGs were taxonomically classified with GTDB‐Tk v1.5.0 with reference data for GTDB R06‐RS202 (Chaumeil et al., 2019). To create a phylogenomic tree, representative genomes from the four families ZC4RG38, *Calditerricolaceae*, *Thermosediminibacteraceae* and *Moorellaceae* were downloaded from GTDB v202 (Parks et al., 2020) using *gtt‐get‐accessions‐from‐GTDB* in GToTree v1.6.12 (Lee, 2019). A concatenated alignment was created using 119 single‐copy genes targeted by the *Firmicutes* HMM profile in GToTree and programs within; (Capella‐Gutiérrez et al., 2009; Eddy, 2011; Edgar, 2004; Hyatt et al., 2010; Lee, 2019; Tange, 2018). The phylogenomic tree was created with IQ‐tree (Nguyen et al., 2015). Substitution models for each partition were selected with ModelFinder (−m MFP) (Kalyaanamoorthy et al., 2017) and support for phylogenetic groups was determined with UFBoot (−bb 1000) (Minh et al., 2013). An *Actinobacteriota* MAG from this study was used to root the tree.

## RESULTS

### Acetate production in heated sediments

**FIGURE 1:**
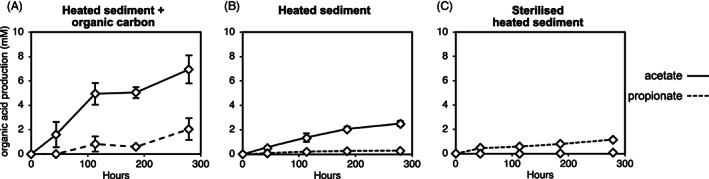
Acetate and propionate production in sediment heated to 80°C. Acetate (solid line) and propionate (dashed line) were measured in anoxic sediment slurries heated to 80°C for 279 h. Sediment slurries were either (A) supplemented with organic carbon; (B) heated without organic carbon amendment; or (C) heated and sterilized with zinc chloride. Error bars show standard deviation of three replicate incubations.

Surface sediments were mixed with anoxic medium and the resulting slurries incubated at 80°C. Sediments were either heated without additional carbon amendment or were supplemented with organic carbon compounds. Heating the sediment resulted in acetate and propionate production in both sets of incubations (Figure 1A,B), with the addition of supplemental organic carbon resulting in higher acetate concentrations being indicative of microbial acetate generation in these incubations.

At high temperature, sediment is increasingly subject to abiotic, thermal transformations of organic matter (Lin et al., 2017; Otte et al., 2018). To confirm that acetate production observed at 80°C was derived from microbial degradation of organic carbon, sediments were also chemically sterilized with zinc chloride prior to incubation. In these sterile controls, acetate production was significantly lower (0.01 mM acetate production) than in unsterilized sediment that was unamended (2.5 mM acetate production) or supplemented with organic substrates (6.9 mM acetate production). Propionate was produced to a similar concentration in both sterilized (Figure 1C) and unsterilized sediments (Figure 1A,B), suggesting propionate results from abiotic, thermal reactions at 80°C. Accumulation of formate (1.0 mM) and lactate (0.6 mM) was also observed. The sterilized control shows that while some acetate can be produced by abiotic, thermal reactions, much higher levels observed in unsterilized sediment is indicative of microbial activity being stimulated at high temperature.

### Temperature‐dependent germination of endospore populations

**FIGURE 2:**
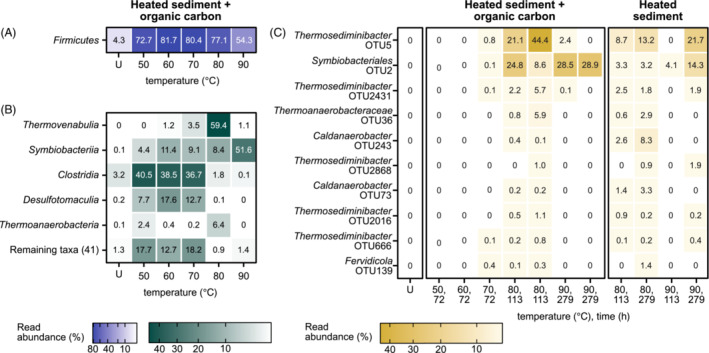
Enrichment of *Firmicutes* in heated sediment. 16S rRNA gene amplicon libraries of sediments heated to 50, 60, 70, 80 and 90°C compared to unheated sediments (‘U’). (A) Read abundance of the endospore‐forming phylum *Firmicutes*. (B) Classes within the *Firmicutes* with the greatest read abundance (top five). (C) OTUs within the *Firmicutes* with the greatest read abundance in sediments heated to ≥80°C (top 10).

16S rRNA gene amplicon libraries were generated from sediments incubated at 50, 60, 70, 80 and 90°C and compared with each other and to amplicon libraries prepared from unheated sediment (Figure 2). The read abundance of endospore‐forming *Firmicutes* increased following heating in all sediment incubations (Figure 2A), indicating that dormant endospore populations in the sediment germinated, resulting in their genomic DNA being extractable from vegetative cells when using standard protocols.

Different populations of endospore‐forming bacteria were detected at 80°C compared to lower temperature incubations. At 80°C, reads from *Firmicutes* were assigned to the classes *Thermovenabulia*, *Symbiobacteriia*, *Thermoanaerobacteria* and *Clostridia* (Figure 2B). Pearson correlation of each operational taxonomic unit (OTU) with acetate accumulation showed high correlation (>0.6) for OTUs from *Thermovenabulia* and *Thermoanaerobacteria* (Figure S1). OTUs from lineages enriched at 80°C were present in lower relative abundances during incubation at 70°C and were not detected at 60 or 50°C (Figure 2C). On the other hand, *Thermosediminibacter* and *Symbiobacteriales* OTUs were also detected at 90°C, suggestive of a hyperthermophilic temperature physiology for these lineages, which are not normally considered to include hyperthermophiles able to grow at temperatures ≥80°C.

### Growth temperature prediction from genomes supports thermophilic activity

**FIGURE 3:**
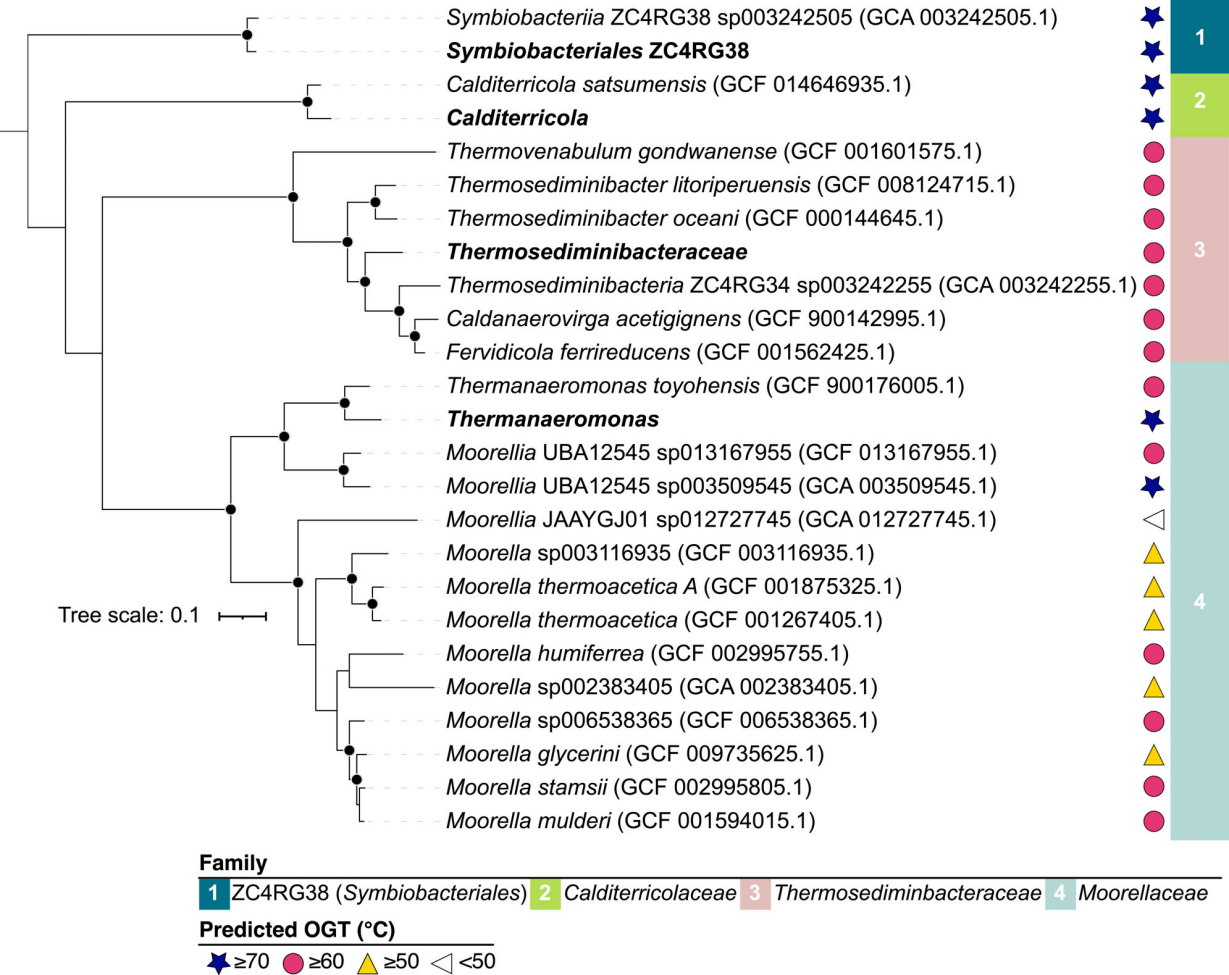
Phylogenomic tree with predicted optimum growth temperature (OGT) of thermophilic endospore‐forming *Firmicutes*. Four thermophile MAGs from this study are shown in bold. Representative genomes from the same families (1) ZC4RG38, (2) *Calditerricolaceae*, (3) *Thermosediminibacteraceae*, and (4) *Moorellaceae* were downloaded from GTDB and included in the tree. The tree is based on 119 concatenated single copy genes. Bootstrap support values ≥99% (1000 replicates) are indicated by filled circles. The scale bar corresponds to per cent average amino acid substitution over the alignment and supports that all four genomes represent novel lineages within their respective phylogenetic groups. Amino acid‐based predictions of OGT were made using Tome (Li et al., 2019).

Two metagenomic datasets were created from sediment slurries incubated at 80°C. Assembled metagenomic contigs were binned and de‐replicated resulting in 13 metagenome‐assembled genomes (MAGs) from four phyla (*Firmicutes* (×4), *Proteobacteria* (×4), *Actinobacteriota* (×4) and *Campylobacterota* (×1); genome completeness is provided in Dataset S1). The optimum growth temperature for each of the 13 MAGs was predicted based on inferred amino acid composition (Li et al., 2019). The four *Firmicutes* MAGs were predicted to have growth temperature optima ranging from 69 to 75°C (Figure 3). *Proteobacteria*, *Actinobacteriota* and *Campylobacterota* were predicted to be mesophiles with temperature optima ranging from 24 to 29°C (Dataset S1). MAGs from microorganisms predicted to be mesophiles were likely binned from relic DNA (Lennon et al., 2018) arising from organisms present in situ that died at elevated temperature and were excluded from further analysis.

Phylogenomic analysis of the four thermophilic *Firmicutes* MAGs showed taxonomic affiliations with the families ZC4RG38 (class *Symbiobacteriales*), *Calditerricolaceae*, *Thermosediminibacteraceae*, and *Moorellaceae* (Figure 3). Comparing these four MAGs with the Genome Taxonomy Database (GTDB) did not uncover any close relatives with >95% amino acid identity (AAI). Closest relatives ranged between 79.9% and 93.3% AAI indicating that these four MAGs represent new genera or species within their respective phylogenetic groups. Genome‐based predictions of optimal growth temperature among related microorganisms included in the phylogenomic tree suggest that the nearest relatives are thermophiles (Figure 3). Accordingly, these populations have been discovered in geothermal subsurface aquifers (Mori et al., 2002; Ogg & Patel, 2009) and high‐temperature compost (Martins et al., 2013; Moriya et al., 2011).

*Symbiobacteriales* and *Thermosediminibacteraceae* MAGs contained 16S rRNA gene sequences that were ≥99% identical to OTUs detected by amplicon sequencing. Based on nucleotide identity, the *Symbiobacteriales* MAG corresponds to OTU2 and the *Thermosediminibacteraceae* MAG corresponds to OTU5 (see OTUs detected at 80 and 90°C in Figure 2C). The 16S rRNA gene sequence from the *Thermanaeromonas* MAG shared greatest nucleotide identity (95%) with OTU36 (Figure 2C). The *Calditerricola* MAG did not include a 16S rRNA gene, but an unbinned *Calditerricola* 16S rRNA gene sequence shared 99.7% nucleotide identity with *Calditerricola* OTU1256 present at only 0.02% read abundance.

### Genomic evidence for sporulation and dormancy

Thermophilic *Firmicutes* detected in ≥80°C sediment incubations are predicted to be endospore formers based on their viable persistence in an environment much below their temperature requirement for growth. The potential for sporulation was confirmed by the presence of core sporulation genes that are conserved in well‐known spore‐forming *Bacilli* and *Clostridia* (Galperin et al., 2012). These include genes required for pre‐septation (Stage 0), post‐septation (Stage II), post‐engulfment (Stages III–VI), spore coat assembly and germination and were present in all *Firmicutes* MAGs (Dataset S2).

### Endospore populations have the metabolic potential for acetate metabolism

**FIGURE 4:**
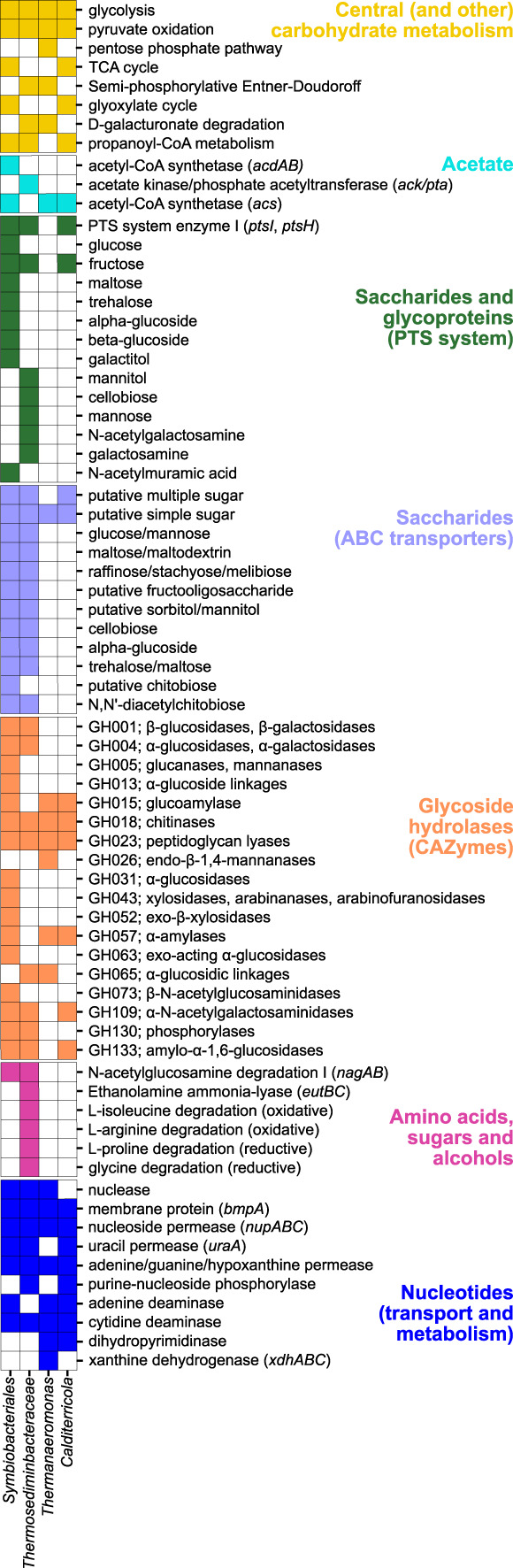
Metabolic potential of thermophilic *Firmicutes*. The presence (filled squares) of select metabolic pathways and genes in four high‐quality MAGs of thermophilic endospore‐forming *Firmicutes*. Details of KEGG modules, CAZymes and MetaCyc pathways used to create the figure are provided in Dataset S3.

*Symbiobacteriales* can produce acetate via ADP‐forming acetyl‐CoA synthetase (*acdAB*) while *Thermosediminibacteraceae* can produce acetate in a two‐step conversion via phosphate acetyltransferase (*pta*) and acetate kinase (*ackA*). Genes from multiple pathways for organic carbon degradation to acetate were present (Figure 4). Both *Symbiobacteriales* and *Thermosediminibacteraceae* possess the phosphoenolpyruvate (PEP):carbohydrate phosphotransferase system (PTS) for uptake and concomitant phosphorylation of carbohydrates (Deutscher et al., 2006). This includes enzyme I (encoded by the *ptsI* gene) and HPr (encoded by the *ptsH* gene) as well as a sugar‐specific enzyme II (EII) permease. Multiple EII permeases and ABC transporters were found (Figure 4) including those for the uptake of plant‐ and algal‐derived saccharides (e.g. cellobiose and mannibiose). Both genomes contained multiple glycoside hydrolases (Figure 4) for the breakdown the glycosidic bonds in carbohydrates producing free fermentable glucose and other monosaccharides.

*Thermanaeromonas* and *Calditerricola* do not harbour genes for acetate production and are therefore unlikely contributors to the acetate production observed in heated sediment incubations. Instead, the presence of AMP‐forming acetyl‐CoA synthetase (*acs*) in both *Calditerricola* and *Thermanaeromonas* genomes suggest these organisms can assimilate acetate.

### Genomic potential to degrade necromass and spore lysates in heated sediment

Cellular necromass contains mostly proteins and amino acids as well as DNA, RNA and membrane sugars. *Thermosediminibacteraceae* has the genomic potential to metabolize amino acids by co‐fermentation using Stickland reactions, with isoleucine and/or arginine as electron donor and proline and/or glycine as electron acceptor (Figure 4). *Thermosediminibacteraceae*, *Symbiobacteriales* and *Thermoanaeromonas* genomes all contain extracellular nuclease for degradation of DNA polymers, and all MAGs contained nucleoside and nucleobase transporters that may be used for uptake of extracellular DNA (Pérez Castro et al., 2021). *Thermanaeromonas* further has the potential to degrade xanthine, a nitrogen‐rich organic compound from nucleic acids that is widespread in aquatic environments (Cunliffe, 2016).

Both *Thermosediminibacterace* and *Symbiobacteriales* have EII permeases for the uptake of membrane sugars (e.g. *N*‐acetyl glucosamine and glucosamine) (Figure 4). Amino sugars phosphorylated by the PTS system then enter glycolysis following the removal of acetyl and amino groups by the enzymes *N‐*acetylgluscosamine‐6‐phosphate deacetylase (*nagA*) and glucosamine‐6‐phosphate deaminase (*nagB*). The *Thermosediminibacteraceae* MAG also contains genes for degradation of ethanolamine, a phospholipid readily available from the degradation of cell membranes.

Genes for dissimilatory sulfite reductase (*dsrAB*), the sulfur relay protein *dsrC* and the electron transport complex *dsrMKJOP* are present in the *Thermanaeromonas* MAG. The absence of sulfate adenylyltransferase (*sat*) and adenylylsulfate reductase (*aprAB*) and presence of thiosulfate reductase (*phsA*) suggests that this organism has the potential to use thiosulfate and/or sulfite. Given that thiosulfate and sulfite are not expected to be present in these experiments, *Thermanaeromonas* presumably grows fermentatively, as reported for the closest related isolate, *T. toyohensis*, in the absence of sulfur compounds (Mori et al., 2002). Alternatively, *Thermanaeromonas* could yield sulfite from the desulfonation of sulfolactate by sulfolactate sulfo‐lyase (*syuAB*), that cleaves *R*‐sulfolactate into pyruvate and sulfite (Denger & Cook, 2010). Sulfolactate is a widespread natural product in plants, algae and prokaryotes and is also a component of bacterial endospores that gets released upon germination (Bonsen et al., 1969; Rein et al., 2005). Sulfolactate produced by germinating endospores could therefore provide a source of organosulfate in heated sediments.

## DISCUSSION

Distinct populations of thermophilic endospore forming bacteria germinate when surface sediments are heated (Figure 2). Thermophilic sulfate reducing bacteria prevail at temperatures ≤70°C, whereas hyperthermophilic organotrophs with the potential to metabolize different pools of organic carbon were selected by heating to 80 and 90°C. When bacteria are provided with multiple carbon sources, easily accessible compounds are selectively metabolized (Deutscher, 2008; Görke & Stülke, 2008). Acetate production in heated sediments supplemented with organic carbon is therefore likely derived from the metabolism of glucose and other easily accessible components. However, acetate was also produced at 80°C without any organic carbon supplement, indicating that components of sedimentary organic matter are accessible to microbial biodegradation by thermophiles and hyperthermophiles.

Organotrophic bacteria enriched here at ≥80°C have the genomic potential to degrade multiple components of sedimentary organic matter. In surface sediments organic matter typically consists of 10%–20% carbohydrates, 10% nitrogenous compounds (mostly amino acids) and 5%–15% lipids, with the remaining fractions consisting of unidentified organic compounds considered to be recalcitrant (Arndt et al., 2013). Genomes of hyperthermophilic endospores contained genes for the degradation of plant and algal‐derived carbohydrates and components of cellular necromass. In this study, sediment was heated to 80°C, which is commonly used as a pasteurization step designed to kill vegetative cells of psychrophilic and mesophilic microorganisms (Hubert et al., 2009). Dead mesophiles formerly prevalent at ambient in situ temperature may therefore offer a source of amino acids, nucleic acids, phospholipids and membrane sugars that can be metabolized by thermophilic organotrophs in surface sediment heating experiments.

Compounds released by germinating endospores provide another potential source of energy. Germinating endospores release the biodegradable compounds sulfolactate (5% dry weight) and dipicolinic acid (5%–15% dry weight) (Bonsen et al., 1969; McClintock et al., 2018; Setlow, 2006). *Thermanaeromonas* was the only spore‐former in this study with the potential to metabolize sulfolactate, whereas all *Firmicutes* MAGs had genes for dipicolinic acid uptake. While anaerobic transformation of dipicolinic acid to acetate, propionate, ammonia and CO_2_ has been shown for a coculture of marine microorganisms (Seyfried & Schink, 1990), an exact enzymatic pathway for dipicolinic acid fermentation has not been elucidated.

Earlier studies that report acetate production upon heating surface or deep sediments have attributed this observation to the temperature activation of organic carbon increasing its bioavailability, thus providing a continuous supply of energy for bacteria and archaea during burial in the deep biosphere (Parkes et al., 2007; Wellsbury et al., 1997). Evidence of microbial acetate production presented here suggests that activation and metabolism of dormant thermophilic endospore populations could also contribute to acetate generation in deep hot sediments. Surface sediments worldwide contain thermophilic endospores (Müller et al., 2014) that via burial seed deeper sediments (Inagaki et al., 2015), where community assembly is driven by selection mechanisms that favour populations adapted to energy limitation (Petro et al., 2017; Starnawski et al., 2017). The resilience of endospores makes these populations well suited to not being filtered out, possibly explaining estimates that endospores account for a significant proportion of microbial biomass in deeper sediments where they have been proposed to outnumber vegetative cells (Lomstein et al., 2012; Wörmer et al., 2019). Buried endospores remain viable (de Rezende et al., 2013; Fang et al., 2017; Lee et al., 2005) and have the potential to germinate in situ when the ambient temperature is high enough, facilitating thermophilic or hyperthermophilic metabolism in deeply buried sediments.

Surface sediments used here and in similar experiments (Parkes et al., 2007; Wellsbury et al., 1997) contain more organic carbon than would be likely encountered in deep, hot marine sediment layers. Degradation of organic matter by shallow organotrophic communities means organic matter availability generally diminishes with depth, such that amino acids from microbial necromass have been proposed as quantitatively the most important organic substrates in deep sediments (Lever et al., 2015; Lomstein et al., 2012; Orsi et al., 2020). Thermophilic and hyperthermophilic spores detected here have the genomic potential to gain energy from microbial necromass and experimental evidence suggests that necromass oxidation occurs widely in the marine environment (Langerhuus et al., 2012; Lomstein et al., 2012; Pérez Castro et al., 2021; Tully et al., 2016; Wasmund et al., 2021). However, available energy from necromass declines with depth such that microorganisms in deep sediments suffer from extreme energy limitation in the absence of residual organic carbon or other energy sources (Bradley et al., 2018; Orsi et al., 2020).

Energy limitation could provide an obstacle for endospores in buried sediments. While our results suggest that temperature is important for activation among specific endospore populations, germination also requires nutrients (or germinants) such as sugars, amino acids, and nucleosides that interact with specific germination receptors in the spore coat (Kochan et al., 2018). Although metabolism of exogenous nutrients (and generation of ATP) is not needed for germination (Setlow et al., 2017), dormant endospores still need to sense conditions that are conducive for growth. On this basis, for buried organotrophic endospores to become active members of the deep hot biosphere, they must not only encounter a suitable temperature, but the surrounding sediments must also have sufficient organic substrates to ensure that germination does not lead to starvation. In this context, the ability to degrade spore components such as dipicolinic acid or sulfolactate, as well as the presence of different enzymes for carbohydrate metabolism in thermophiles relative to mesophiles (Hubert et al., 2009), are features that could contribute to the fitness of spore‐forming bacteria in the deep biosphere.

Sediment heating experiments offer useful models for making predictions about microbial ecology in the deep hot subsurface, where sampling is difficult and biomass levels are generally too low to facilitate direct metagenomic studies. Sediments harbour a seed bank of endospores with genomic and functional diversity and that can remain viable for long periods of time to enable their selection when environmental circumstances are appropriate. Our results offer insight into the metabolic potential of thermophilic and hyperthermophilic organotrophs with the potential to germinate in deep, hot environments where organic carbon, necromass or metabolites released during germination could contribute to their organotrophic metabolism as vegetative cells.

## CONFLICT OF INTEREST

The author declares that there is no conflict of interest that could be perceived as prejudicing the impartiality of the research reported.

## Supporting information


**Appendix S1** Supplementary Information

## Data Availability

Organic acid data from in this study are provided in the Supplementary Information (Dataset S4). Sequence data have been deposited in GenBank under the BioProject PRJNA371432. BioSample metadata for MAGs described in this study are available in the NCBI BioSample database (http://www.ncbi.nlm.nih.gov/biosample/) under accession numbers SAMN22252053–SAMN22252056.
