# Supplementary material for: Hyperthermophilic endospores germinate and metabolize organic carbon in sediments heated to 80°C
Source: Environ Microbiol. 2022 Sep 13;24(11):5534–45. doi: 10.1111/1462-2920.16167 (PMC9826295; doi:10.1111/1462-2920.16167)
Supplement: Supplementary file 1 — Appendix S1 Supplementary Information [file EMI-24-5534-s001.zip › EMI_16167_Supporting_Information.pdf]

Supporting Information

Hyperthermophilic endospores germinate and metabolise organic carbon in sediments heated to 80°C

Emma Bell[1*], Jayne E. Rattray[1], Kathryn Sloan[1], Angela Sherry[2], Giovanni Pilloni[3], Casey R. J. Hubert[1]

[1]Geomicrobiology Group, Department of Biological Sciences, University of Calgary, Calgary, Alberta, Canada

[2]Hub for Biotechnology in the Built Environment, Department of Applied Sciences, Northumbria University, Newcastle upon Tyne, United Kingdom

[3]ExxonMobil Research and Engineering, Annandale, NJ, USA

Supplementary Datasets

Dataset S1: Dereplicated MAGs.
Dataset S2: Sporulation genes present in *Firmicutes* MAGs.
Dataset S3: Presence/absence of select metabolic pathways.
Dataset S4: Organic acid data.

Supplementary Figures

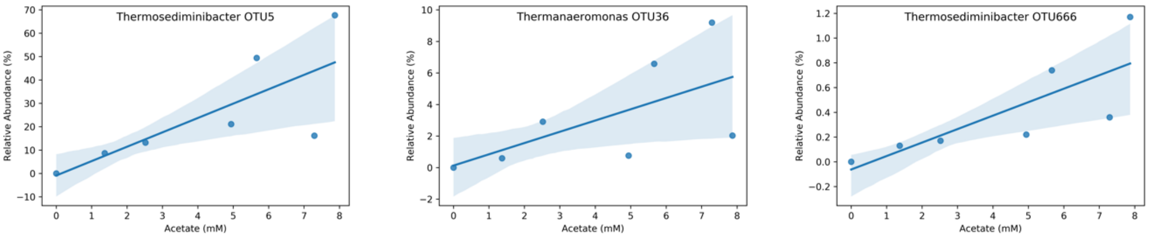

Figure S1: Normalised read abundance of OTUs corresponding to acetate production at different sampling time points. *Firmicutes* OTUs from Figure 2C in the main text that were positively correlated (Pearson correlation >0.6) with acetate were selected for visualisation. The shaded area shows the 90% confidence interval.
